# Early onset of clinical leishmaniosis in a litter of pups with evidence of in utero transmission

**DOI:** 10.1186/s13071-021-04824-0

**Published:** 2021-06-15

**Authors:** Harold Salant, Yaarit Nachum‑Biala, Barbara Feinmesser, Maya Perelmutter, Gad Baneth

**Affiliations:** 1 Koret School of Veterinary Medicine, Hebrew University of Jerusalem, Rehovot, Israel; 2 Small Animal Veterinary Clinic, Hanadiv 7, Herzliya, Israel

**Keywords:** Canine, Israel, *Leishmania infantum*, Litter

## Abstract

**Background:**

Canine leishmaniosis (CanL) is a zoonotic disease caused by *Leishmania infantum*. Although usually transmitted by phlebotomine sand flies, infection by vertical transmission and by blood transfusion have also been reported.

**Methods:**

We describe the very early onset of clinical leishmaniosis, starting from 2 months of age, in a litter of pups born to an infected dam and sire. Seven pups from the litter of nine living in different households showed alopecic, exfoliative dermatitis and ulcerative cutaneous lesions. All pups and both parents were tested on at least one occasion both serologically, by enzyme-linked immunosorbent assay (ELISA), and by polymerase chain reaction (PCR) targeting the *Leishmania* ribosomal operon internal transcribed spacer 1 region and a short fragment of the kinetoplast minicircle; positive amplicons were sequenced.

**Results:**

All nine pups were PCR positive for *L. infantum* verified by DNA sequencing, seven were positive by conjunctival, five by blood, four by lymph node, and one by skin PCR from an ulcerative lesion. Both pups with no clinical signs were seronegative, while five of the seven pups with dermatologic abnormalities were seropositive by ELISA. The sire had typical clinical dermatologic and visceral findings of CanL, was seropositive and PCR positive for *L. infantum* in the lymph node and fluid from the vas deferens tested after the testes were removed by castration. The dam was sub-clinically infected and seronegative, but positive by blood, lymph node and conjunctival PCR for *L. infantum.* Allopurinol administered to all clinically affected dogs resulted in clinical recovery.

**Conclusions:**

Infection with *L. infantum* in both parents, the very early age of clinical onset among most of the pups, and the fact that the puppies were born and detected with signs of leishmaniosis in the winter, which is a season without sand fly activity in Israel, strongly suggest vertical transmission. Awareness of the possibility of vertical transmission of *L. infantum* and infection in littermates should be increased. It is recommended that littermates of young dogs with clinical leishmaniosis should be tested for sub-clinical infection as they may also be infectious to sand flies and thus to other dogs and to humans. Restricting the mating of infected bitches should also be considered to prevent the vertical transmission of the infection.

**Graphic abstract:**

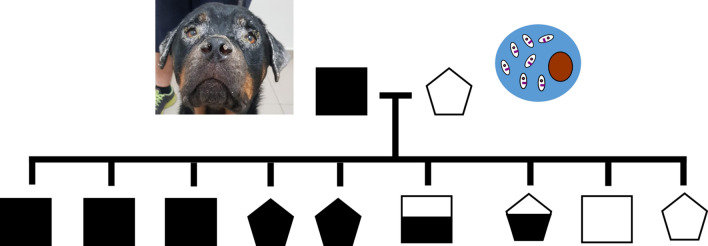

## Background

Visceral leishmaniasis is a widespread zoonotic disease of major public health and veterinary importance that occurs on all the continents except for Oceania [1]. It is caused by a protozoon, *Leishmania infantum*, which belongs to the genus *Leishmania,* and is grouped into the *Leishmania donovani* complex, which includes *Leishmania donovani* and *Leishmania infantum*. The natural hosts of *L. infantum* include dogs, humans, cats and wildlife [2]. Dogs are considered the most important peridomestic source for human infection [3]. Infection in dogs may result in a wide spectrum of manifestations ranging from an absence of clinical signs to multisystemic disease with clinical signs varying from poor body condition, generalized muscular atrophy, lymphadenomegaly and excessive skin scaling to severe hematological, renal, ocular, bone and joint disease.

Transmission of *Leishmania* among canids and to humans occurs through the bite of infected sand flies that belong to the genus *Phlebotomus* in the Old World and the genus *Lutzomyia* in the New World. However, transmission in the absence of the vector has been reported, including infection due to blood transfusion [4, 5], vertical transmission in the absence of known suitable biological vectors [6–8], and venereal transmission from the semen of naturally infected males to susceptible bitches [9, 10]. The incubation period after sand fly infection can extend from 3 months to 7 years [11], and clinical cases of leishmaniosis of pups younger than 5 months of age are extremely rare [12–14].

This study describes *L. infantum* infection in a litter of nine young pups, their dam and sire, which suggests in utero infection, and demonstrates the early onset of disease.

### Clinical study

Nine newly adopted 2—month-old mixed Rottweiler pups including four females and five males from the same litter were taken to two veterinary clinics in central Israel for routine vaccination during March and April 2019. The owners reported lesions on their dogs during the vaccination period and on subsequent visits

**Fig. 1a–c:**
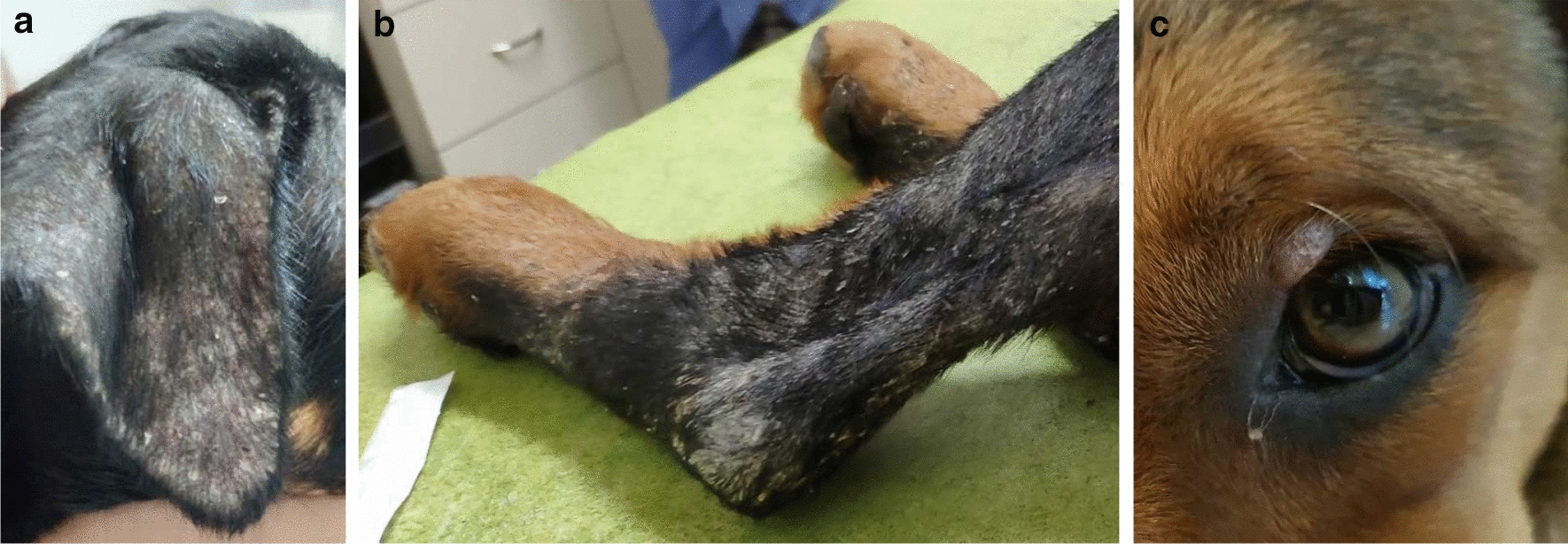
Exfoliate dermatitis in the puppies. **a** Alopecia and exfoliative dermatitis on the ear of pup no. 1, **b** exfoliative dermatitis on the hind leg of pup no. 1, **c** mild exfoliative dermatitis on the left eyelid of pup no. 2

**Fig. 2:**
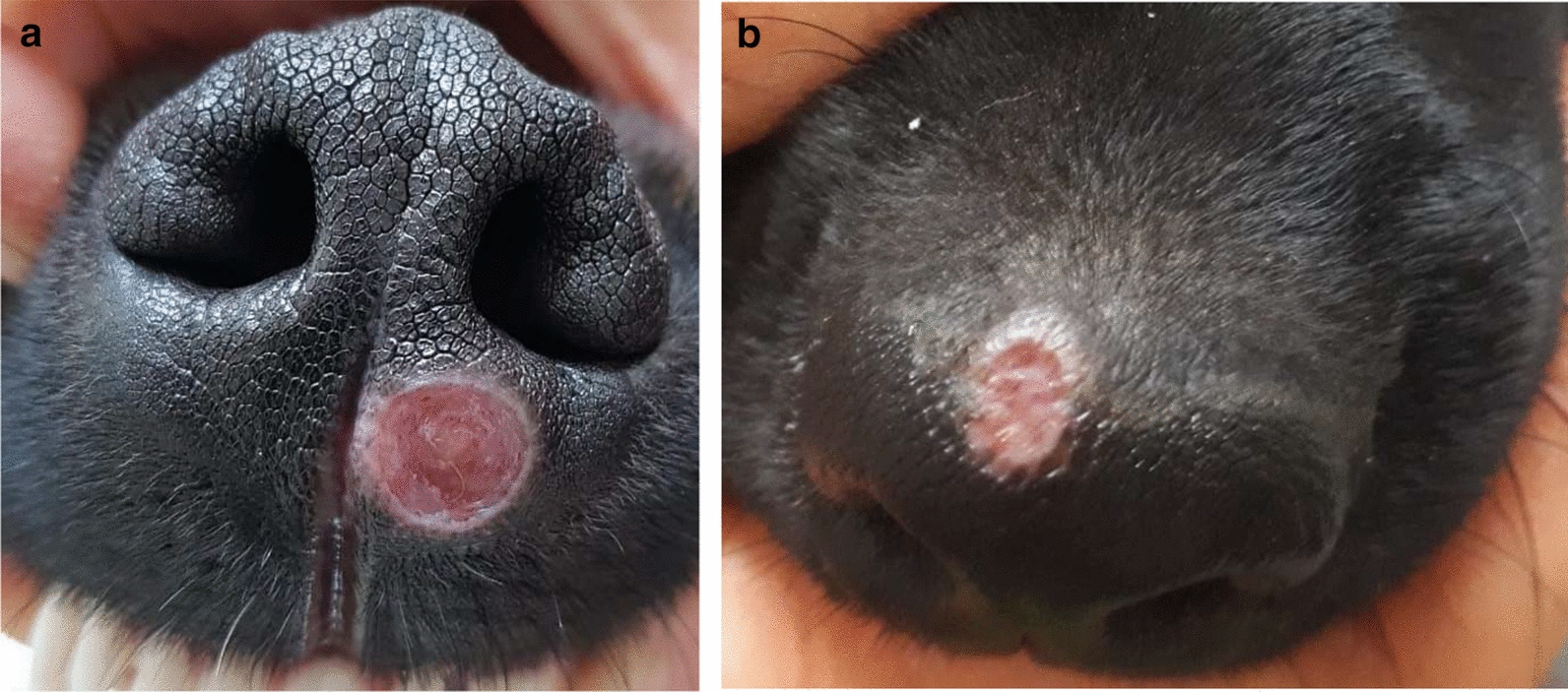
Ulcerative lesions on the lower muzzle of pup no. 7 (**a)** and on the upper muzzle of pup no. 5 (**b**)

**Fig. 3:**
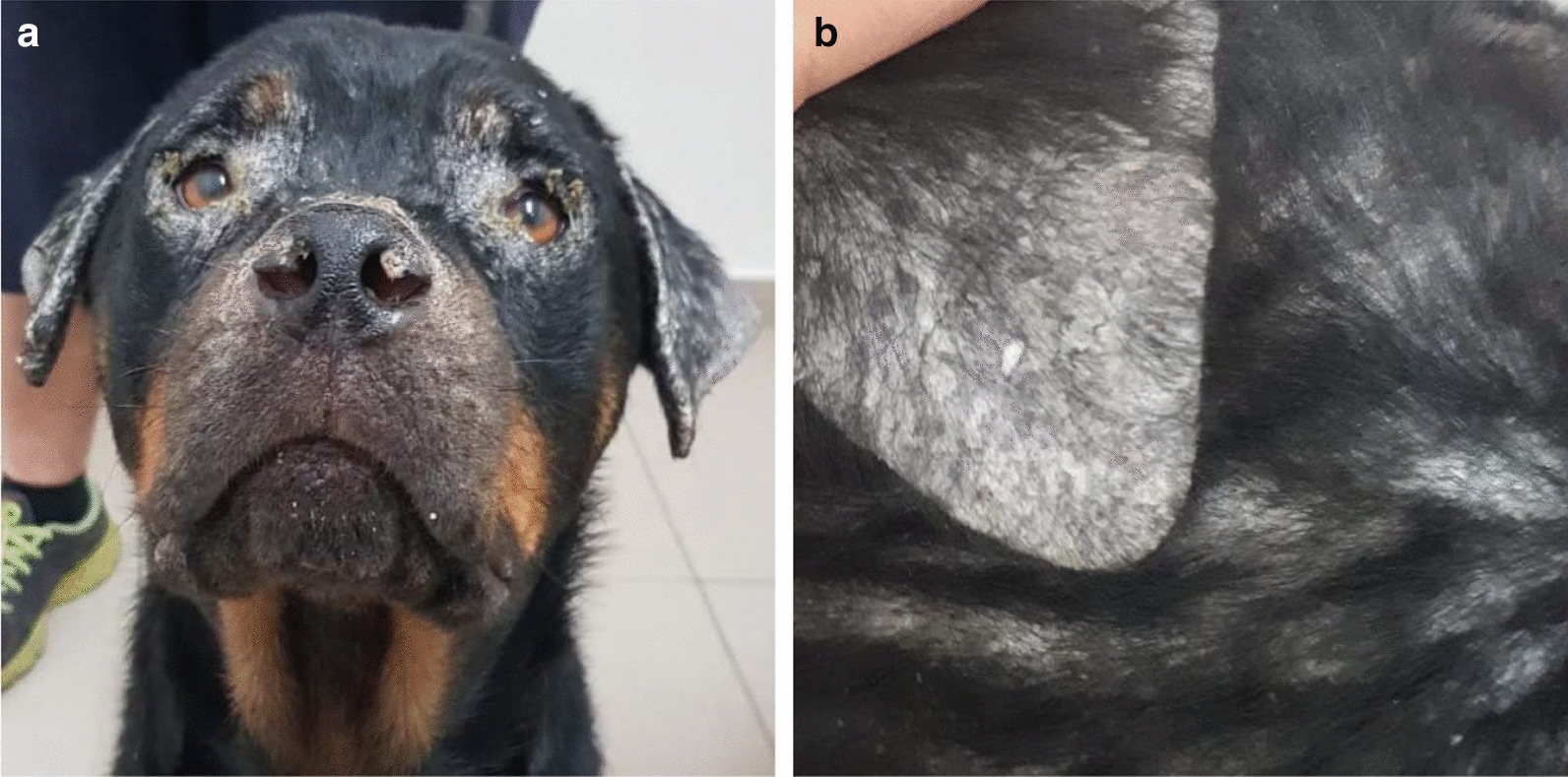
The sire of the litter presenting with exfoliative dermatitis typical of canine leishmaniosis over the face and around the eyes (**a**) and over the ear (**b**)

The lesions on the affected pups varied from localized to generalized exfoliative dermatitis on the face, around the eyes, on the ears, pinna, back, elbows, and nasal dorsum (nos. 1 and 2) (Fig. 1; Table 1) and also included ulcerative lesions (nos. 5 and 7) (Fig. 2). In addition to dermatitis, two pups had conjunctivitis (nos. 1 and 5), one of the pups had fever and peripheral lymphadenomegaly with lameness (no. 1) and one had signs of diarrhea and vomiting (no. 5; Table 1). The owner of the 2.5-year-old male Rottweiler that had sired the pups had also consulted with the same clinic concerning the bilateral ocular purulent discharge as well as multifocal alopecic, scaly cutaneous lesions that his dog presented with approximately 3 months previously and were compatible with canine leishmaniosis (CanL) (Fig. 3a, b). Since the clinical appearance of the cutaneous lesions of the pups and their sire resembled leishmaniosis, and Israel is endemic for canine *L. infantum* [15], it was decided to do further testing for this disease and also to rule out differential diagnoses. The dam that whelped the pups was a 1.5-year-old mixed-breed medium-sized bitch located in a different city to where the pups were living after adoption, and was apparently free of any clinical signs suggestive of leishmaniosis. In addition to the seven pups with dermal lesions, the two other pups, a male and female (nos. 8 and 9), from the same litter, were apparently healthy and free of dermal lesions. The dam and these two pups were also examined physically and evaluated for the presence of suspected sub-clinical infection with *L. infantum*.

**Table 1:** Clinical manifestations, gender, age at onset of clinical signs, serology, polymerase chain reaction (*PCR*), laboratory abnormalities, therapy and response to therapy among individual dogs of a cluster of canine leishmaniosis caused by *Leishmania infantum*

Dog identity	Gender	Clinical signs	Age of onset of clinical signs in months	ELISA serology	PCR positivity and loci^a^	PCR-positive tissue	Hematological and biochemical changes	Treatment	Response to treatment
Puppy 1	Female	Nasal, truncal and pinnal crusty exfoliative dermatitis; peripheral lymphadenomegaly; keratoconjunctivitis; fever; right hind limb lameness	2	Positive	kDNA, ITS1		Leukocytosis; hyperproteinemia; hypergammaglobulinemia; increased liver enzymes; normochromic, normocytic anemia	Allopurinol	Clinical remission within 3 months
Puppy 2	Female	Nasal alopecia and mild exfoliative periocular dermatitis	2.5	Positive	kDNA, ITS1	Lymph node, blood, conj. Lymph node, blood	No abnormalities	Allopurinol	Clinical remission within 3 months
Puppy 3	Male	Pinnal and periocular exfoliative dermatitis	2.5	Positive	kDNA	Blood, conj.	No abnormalities	Allopurinol	Clinical remission within 3 months
Puppy 4	Female	Localized alopecia of nasal planum	2.5	Negative	ITS1	Conj.	No abnormalities	Allopurinol	Clinical remission within 3 months
Puppy 5	Male	Localized nasal ulcer; diarrhea and vomiting; conjunctivitis	2.5	Positive	kDNA, ITS1	Lymph node, conj.	Hypergammaglobulinemia	Allopurinol	Clinical remission within 3 months
Puppy 6	Male	Pinnal alopecia	3.5	Positive	kDNA, ITS1	Lymph node, blood, conj.	Hyperproteinemia; hypergammaglobulinemia	Allopurinol	Clinical remission within 3 months
Puppy 7	Male	Cutaneous nasal planum ulcer	6	Negative	kDNA, ITS1	Skin	No abnormalities	Allopurinol	Clinical remission within 3 months
Puppy 8	Female			Negative	kDNA, ITS1	Blood, conj.	No abnormalities		
Puppy 9	Male			Negative	kDNA, ITS1	Conj.	No abnormalities		
Sire	Male	Exfoliative dermatitis; keratoconjunctivitis; weight loss, weakness	25	Positive	kDNA	Lymph node, semen	Not performed	Allopurinol	Clinical remission within 3 months
Dam	Female			Negative	kDNA, ITS1	Lymph node, blood, conj.	No abnormalities		

*ELISA* Enzyme-linked immunosorbent assay,* conj*. conjunctival swab

^a^Targeted loci found to be PCR positive; PCR targeting the *Leishmania* kinetoplast minicircle (*kDNA*); *Leishmania* ribosomal operon internal transcribed spacer 1 (*ITS1*) region

## Methods

Multiple skin scrapings from lesions of the seven pups with dermal lesions were performed and samples were evaluated for *Demodex* spp. and *Sarcoptes scabiei* mites by microscopy. In addition, dermatophyte cultures were performed for two of the samples with Dermatophyte Test Medium (Biopronix, Biogal, Kibbutz Gilad, Israel).

Blood samples were collected from the cephalic vein and samples were sent for hematology, serum biochemistry, PCR and serology for *Leishmania*. Conjunctival swabs were taken from both eyes of each dog, and popliteal lymph node aspirates of animals with palpable lymph nodes were submitted for PCR. A PCR was also performed for a skin biopsy of a puppy (no. 7) that presented with a single ulcer of the nasal planum.

Serology for anti-leishmanial antibodies was performed by enzyme-linked immunosorbent assay using *L. infantum* antigen, as described previously [16]. DNA was extracted from 200 µl of ethylenediaminetetraacetic acid-anticoagulated blood and from lymph node aspirates of the dogs using the illustra blood genomicPrep Mini Spin Kit (GE Health Care, Buckinghamshire, UK). DNA was extracted from conjunctival swabs using the Quick-DNA Miniprep Plus Kit (Zymo Research, USA). *L. infantum* detection was performed by real-time PCR using primers JW11/JW12 targeting a 120-bp sequence of the *Leishmania* short fragment of the kinetoplast minicircle [16, 17]. Additional detection and identification were carried out by PCR using primers ITS-219F and ITS-219R to amplify a 265-bp fragment of the *Leishmania* ribosomal operon internal transcribed spacer 1 (ITS1) region, and then evaluation was carried out by high resolution melt analysis (*Leishmania* ITS1 high resolution melt PCR) [18]. PCR was performed using the StepOnePlus real-time PCR thermal cycler (Applied Biosystems, Foster City, CA) as previously described [19]. DNA extracted from a promastigote culture of *L. infantum* was used as the positive control for PCR, and DNA from colony-bred dogs negative by PCR for vector-borne pathogens including *L. infantum* was used as a negative control. A non-template control with the same reagents described above but without DNA was added to each PCR to rule out contamination. Positive DNA amplicons were purified (EXO-Sap; New England Biolabs, Ipswich, MA) and sequenced at the Center for Genomic Analyses at the Hebrew University (Jerusalem, Israel) using the BigDye Terminator cycle on an Applied Biosystems ABI3700 DNA Analyzer. The ABI Data Collection and Sequence Analysis software (ABI, Carlsbad, CA) was used for analysis. All amplicons from PCR in this study underwent sequencing to verify infection with *L. infantum*. DNA sequences were compared to other sequences deposited in GenBank using the BLASTn website hosted by the National Center for Biotechnology Information (NCBI), National Institutes of Health, USA (http://www.ncbi.nlm.nih.gov).

## Results

**Fig. 4:**
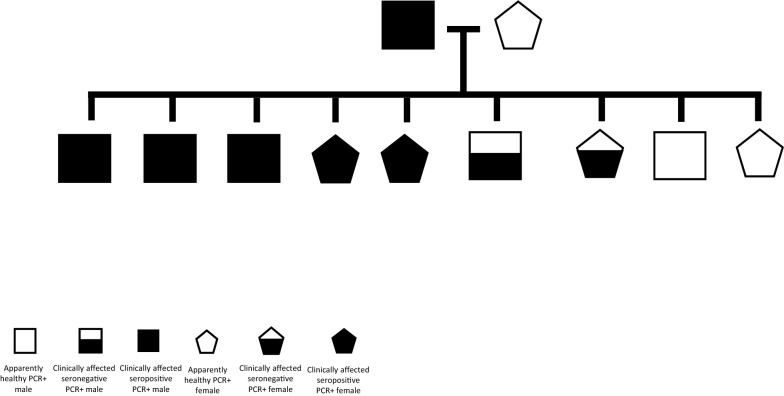
Distribution of outcomes of *Leishmania infantum* infection of the nine pups and their parents indicating which had clinical disease, which were PCR positive and which were seropositive

All nine pups were PCR positive. Seven were positive by conjunctival, five by blood, four by lymph node, and one by skin PCR for an ulcerative lesion (no. 7) (Table 1; Fig. 4). PCR was undertaken for the skin of pup no. 7 since it was negative by PCR for other tissues. Five of the nine pups were seropositive. Both apparently healthy pups were seronegative, while five of the seven pups with dermatologic abnormalities were seropositive. Only three pups had clinicopathological abnormalities (nos. 1, 5, 6), and they were also seropositive.

The sire, which had typical clinical signs of CanL including facial and ear alopecia, exfoliative dermatitis, purulent keratoconjunctivitis, lymphadenomegaly, weight loss and lethargy (Fig. 3), was seropositive, and PCR positive, for *L. infantum* in the lymph node. A year after the sire was diagnosed with CanL it was castrated; during this procedure, fluid from the vas deferens was obtained, which tested positive by PCR for *L. infantum*. The dam, on the other hand, did not show signs of disease, and was seronegative. However, the dam was positive by blood, lymph node and conjunctival PCR for *L. infantum.* All skin samples were negative when tested by fungal dermatophyte culture and for ear mites.

The sire and pups with clinical signs of CanL were treated with allopurinol (Alloril 100; Dexcell, Israel) at 10 mg/kg by mouth twice daily. The dogs were examined clinically at follow-ups during the treatment period, when serological and PCR tests were repeated.

**Fig. 5:**
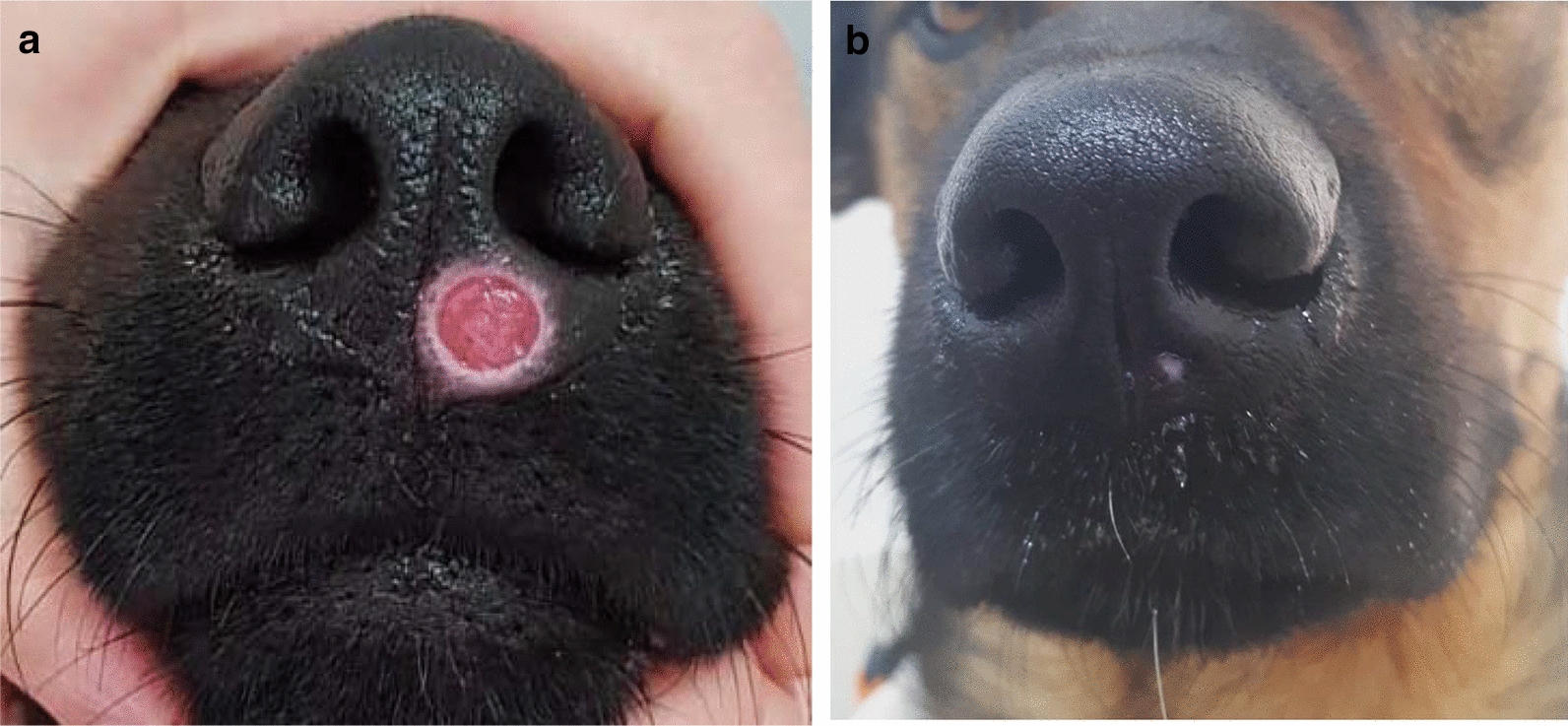
**a, b** Response to allopurinol treatment. **a** Ulcer on muzzle of pup no. 7 before treatment, **b** healing of ulcer on the muzzle of pup no. 7 after 2.5 months of allopurinol treatment (note the small depigmented scar on the muzzle)

Allopurinol, which was administered to all clinically affected dogs, resulted in an improvement in dermatological abnormalities within 3 weeks. All affected pets were maintained on allopurinol treatment, and the owner of each pup was advised that their dog should wear an insecticide-impregnated collar to prevent sand fly bites, and to repeat their dog’s blood tests every 6 months for *Leishmania* serological monitoring. Treatment resulted in clinical recovery of all affected animals within 3 months (Fig. 5; Table 1); treatment had been continuously administered for over 1 year when this report was composed, with all affected dogs remaining in clinical remission. Repeated tests indicated that the serologically positive pups and sire remained *Leishmania* seropositive, albeit at lower titers following treatment, while the seronegative pups remained seronegative.

## Discussion

This report provides a rare description of CanL with the very early onset of clinical signs in a litter of pups. Four of the nine pups had typical facial dermatitis at 2 and 2.5 months of age and a fifth pup showed facial dermatitis at 3.5 months of age, with some pups more severely affected than others. While all the apparently healthy pups were seronegative, two clinically affected PCR-positive pups were also seronegative. This is an interesting finding, as in utero infection may result in the modulation of anti-parasite immune responses in the fetus and neonate [20]. However, intervals for seroconversion in naturally infected dogs can range from 1 to 22 months (median 5 months), and 1–6 months (median, 3 months) for experimentally infected dogs [21]. The sire of the litter, a Rottweiler that developed typical dermal abnormalities, belongs to a breed known to be very susceptible to the development of clinical leishmaniosis [13, 22, 23]. Thus, the phenotypic similarity of some of the pups with the sire may explain why they were more affected by the disease (Fig. 1a, b).

The onset of clinical CanL after exposure to infectious sand fly bites is usually slow, and the appearance of clinical signs under natural conditions has been reported to take place only after a long time lag from infection. In one study where 43 naive dogs were exposed to infectious sand flies in an endemic area in southern Italy, the first dog showed clinical signs of leishmaniosis 10 months after exposure [12]. In a study of 390 CanL cases treated at the Autonomous University of Barcelona, Spain, the youngest case was 6 months old [13]. In the current study, pups already displayed cutaneous signs of CanL between 2 and 2.5 months of age at their initial visit to their veterinarian for vaccination.

This early age at which the pups showed clinical disease in conjunction with the confirmation of the disease in both parents and in the sire’s semen, and the fact that the pups were born and found to have leishmaniosis in the winter, which is a season when sand flies are not active in Israel [24–26], strongly suggest vertical transmission of the parasite from the mother to her pups by transplacental transmission, and perhaps initial, venereal transmission from the sire to the dam [7, 9, 10]. The lack of clinical signs suggestive of *L. infantum* in the dam and seronegativity of the dam are of little significance as the frequency of transplacental transmission does not differ between clinically affected and apparently healthy pregnant bitches, and the clinical status of a bitch is not predictive of the potential for transplacental transmission of *Leishmania* [27]. Other studies have reported evidence of in utero transmission of *Leishmania* to pups of naturally infected bitches. Stillborn pups studied in Brazil showed evidence of transplacental transmission determined by PCR and immunohistochemistry [27, 28]. Studies from the USA [29, 30] and Europe [7, 8], or in experimentally infected bitches [31, 32], have also presented evidence of transplacental transmission of *Leishmania*. Most of these previous studies have based their in utero evidence on observation of gross or histopathological changes, and observation of parasites or confirmation of infection by PCR in aborted or neonatal pup tissue. However, a study by Gibson-Corley et al. [29] reported evidence of in utero transmission after pups born to a *L. infantum*-seronegative bitch in a non-endemic region developed seropositivity and clinical signs at 19 months of age.

Although the results of the present study are suggestive of vertical transmission, it is challenging to prove this because the litter was born in a country where leishmaniosis is endemic and the pups could have been exposed to infectious sand fly bites. Despite this, it is difficult to explain how clinical signs of leishmaniosis were present in 2- to 2.5-month-old pups when other studies have shown that when the disease develops following sand fly transmission it requires a substantially longer time to present clinically [12–14].

Similar to findings reported by others [22, 33, 34], three of the infected dogs displayed hematological and biochemical changes including leukocytosis and monocytosis, hypergammaglobulinemia and increased liver enzyme activities (Table 1). Creatinine levels of all the affected pups were within the normal range, suggesting that the disease had not progressed to the stage of severe renal injury. One puppy (no. 7) that presented with a suspicious cutaneous nasal ulcer was only PCR positive for *L. infantum* on a sample collected from a cutaneous ulcerative lesion, without any serological indication of infection. This is an unusual presentation for *L. infantum* infection, as dogs with clinical signs are usually seropositive [22, 35].

Three dogs, the dam of the litter and two of her pups (nos. 8 and 9), none of which had clinical signs suggestive of leishmaniosis, and all of which were *Leishmania* seronegative, were found to be PCR positive. Sub-clinical infection with *L. infantum* is frequently observed and therefore these results could be expected, especially in dogs that produce a successful immune response to infection and are able to control the infection [36]. On the other hand, two of the pups with CanL infection demonstrated by PCR positivity and clinical disease (nos. 4 and 6) were seronegative. Clinical signs of leishmaniosis are associated with seropositivity and usually with a high titer [35]. The phenomenon of disease with seronegative findings may be related to in utero infection with tolerance of the immune response to fetal infection; therefore, despite infection, no demonstrable antibody titers are found. A study on the immunologic progression of CanL in three littermates naturally infected in utero in North America that were followed for 6 years indicated that each dog had a different pattern of response to infection: one dog that was both seropositive and PCR positive developed clinical disease; a second dog that was clinically healthy was seropositive and PCR positive; while the third dog was clinically healthy, PCR positive and never developed an antibody titer that was above the cut-off titer [37]. That study showed that intrauterine infection with *L. infantum* may lead to littermates presenting with a variety of clinical manifestations, as found also in the pups in our study.

Overall, the pups in the present study can be divided into groups according to their response to infection: (i) apparently healthy, seronegative, PCR positive (nos. 8 and 9); (ii) clinically affected, seronegative, PCR positive (nos. 4 and 7); (iii) clinically affected, seropositive, PCR positive (nos. 1, 2, 3, 5, and 6) (Fig. 4; Table 1). These groups represent the different immune and inflammatory responses that pups may produce due to *L. infantum* infection at an early age, which appear to differ from the responses of older dogs that are infected by sand fly bites and rarely develop seronegative infection with clinical signs as seen in two of nine pups in this study. Different responses have also been found in pups affected by other in utero protozoal infections such as *Neospora caninum*, where some littermates may develop severe clinical disease, whereas others may only be seropositive or have no signs of infection [38, 39].

All the affected dogs recovered clinically within 3 months after the commencement of allopurinol treatment (Table 1). Therapy with anti-leishmanial drugs such as allopurinol often leads to clinical cure [40], and the majority of dogs experience clinical improvement within the first month of therapy, unless they have severe kidney disease due to leishmaniosis or are at an advanced stage of the disease [22]. A longer period of therapy is required for more severe cases before initial clinical improvement is apparent [22, 41].

## Conclusions

In conclusion, we describe a cluster of clinical leishmaniosis amongst a sire and seven of nine littermates, and subclinical *L. infantum* infection in the two other pups and the dam. Leishmaniosis in both parents and the early age at clinical onset of most of the pups strongly suggest vertical transmission. Awareness of the possibility of vertical transmission and infection in littermates should be increased. It is recommended that the littermates of young dogs with clinical signs of leishmaniosis should be examined for infection as they may also transmit the infection to sand flies and thus to other dogs and to humans. Restricting the mating of infected bitches should also be considered to prevent the vertical transmission of *L. infantum*.

## Data Availability

All data generated or analyzed during this study are included in this published article.

## Abbreviations

ELISA: Enzyme-linked immunosorbent assay
ITS1: Ribosomal operon internal transcribed spacer 1 region
kDNA: Short fragment of the kinetoplast minicircle
NCBI: National Center for Biotechnology Information
PCR: Polymerase chain reaction
